# Forty-Fourth Annual Report of the Friends’ Asylum, near Philadelphia

**Published:** 1861-06

**Authors:** 


Forty-fourth Annual Report on the State of the Asylum for the Relief of Persons Deprived of the Use of their Reason.—Published by Direction of the Contributors, Third Month, 1861, Philadelphia.

